# Modulation of Alloimmunity by Heat Shock Proteins

**DOI:** 10.3389/fimmu.2016.00303

**Published:** 2016-08-09

**Authors:** Thiago J. Borges, Benjamin J. Lang, Rafael L. Lopes, Cristina Bonorino

**Affiliations:** ^1^Faculdade de Biociências e Instituto de Pesquisas Biomédicas, Pontifícia Universidade Católica do Rio Grande do Sul, Porto Alegre, Rio Grande do Sul, Brazil; ^2^Department of Radiation Oncology, Center for Life Sciences, Beth Israel Deaconess Medical Center, Harvard Medical School, Boston, MA, USA

**Keywords:** Hsps, transplantation, alloimmunity, dendritic cells, immune regulation

## Abstract

The immunological mechanisms that evolved for host defense against pathogens and injury are also responsible for transplant rejection. Host rejection of foreign tissue was originally thought to be mediated mainly by T cell recognition of foreign MHC alleles. Management of solid organ transplant rejection has thus focused mainly on inhibition of T cell function and matching MHC alleles between donor and host. Recently, however, it has been demonstrated that the magnitude of the initial innate immune responses upon transplantation has a decisive impact on rejection. The exact mechanisms underlying this phenomenon have yet to be characterized. Ischemic cell death and inflammation that occur upon transplantation are synonymous with extracellular release of various heat shock proteins (Hsps), many of which have been shown to have immune-modulatory properties. Here, we review the impact of Hsps upon alloimmunity and discuss the potential use of Hsps as accessory agents to improve solid organ transplant outcomes.

## Introduction

During organ transplantation, tissues are injured as a consequence of ischemia and reperfusion. Upon organ harvest, ischemia ensues and continues during organ preservation. Reperfusion happens upon anastomosis of graft vessels. Ischemia–reperfusion injury (IRI) induces cell death by necrosis and apoptosis leading to production of molecules denominated damage-associated molecular pattern (DAMPs) or alarmins (1). These are self, intracellular molecules, which are released by injured or necrotic cells under pathological conditions. In the extracellular environment, they can interact with and activate innate immune cells, acting as “danger signals” (2, 3). Resident macrophages and dendritic cells (DCs) are sensitive to DAMPs signals. These cells sense initial ischemic insults through pattern recognition receptors (PRRs). The engagement PRRs, including toll-like receptors (TLRs) and scavenger receptors (SRs), trigger intracellular signaling cascades that culminate in activation of transcription factors, which coordinate production of inflammatory cytokines and chemokines, upregulation of MHC and co-stimulatory molecules. DCs are the major antigen-presenting cells (APCs) and once activated in tissues, migrate to draining lymph nodes, and stimulate alloreactive T cell responses. DCs have to deliver three signals for optimal activation of T cells: (i) the expression of peptide:MHC complexes that will be recognized by the T cell receptor (TCR); (ii) co-stimulatory molecules; and (iii) cytokines that will shape T cell-mediated responses. Macrophages are also important contributors to IRI-induced inflammation and produce immune-stimulatory cytokines TNF-α, IL-1β, IL-6, and MCP-1, and chemokines which facilitate recruitment of alloreactive T-cells to the graft site (4, 5). While alloantigen-specific T cells are responsible for subsequent organ rejection and destruction, this outcome is very much shaped by the local inflammatory state of the graft site (6). Thus, alloreactive immune responses result from activation of cellular components of both innate and adaptive immunity (7).

To improve the longevity of solid organ transplants, it is desirable to develop methods to limit IRI-induced inflammation and the ensuing alloreactive immune response. This review will discuss how heat shock proteins (Hsps) can modulate inflammatory and allogeneic immune responses, and how this can be harnessed to manage graft rejection in solid organ transplantation.

## The Heat Shock Response and Cytoprotective Properties of Hsps

The heat shock response is universal and conserved from bacteria to mammals. It can be triggered by a range of protein-damaging conditions that affect organisms such as heat, anaerobiosis/hypoxia, oxidative stress, inflammation, fever, and infection. Activation of the heat shock response is marked by upregulation of various Hsps. Under stress conditions, intracellular Hsps prevent protein aggregation, refold damaged proteins, and target damaged proteins for degradation. Under non-stress conditions, Hsps assist the folding of recently synthetized proteins, the translocation of proteins between organelles, and also regulate the cell cycle. These homeostatic functions of intracellular Hsps support proteome integrity and thereby promote cell viability. In addition, Hsps, such as Hsp70 and Hsp27, have been shown to negatively regulate multiple apoptotic signaling events including mitochondrial cytochrome *c* release (Hsp27), mitochondrial release of Smac (Hsp27), nuclear translocation of apoptosis-inducing factor (AIF) (Hsp70), and cleavage of procaspase 3 (Hsp70) (8–12). Hsps are conventionally grouped into families according to their molecular weight (e.g., Hsp40, Hsp70, and Hsp100) (13, 14). Functional cooperation exists between family members; however, individual Hsp species perform distinct functions that can also be context dependent. In the context of IRI and organ transplantation, increased Hsp levels have been associated with cytoprotection, improvement of organ viability, and function after ischemia–reperfusion (15).

## Intracellular Hsps Protect Allografts from Ischemia–Reperfusion Injury and Improve Graft Survival

Increased levels of Hsps in transplant organ cells either by treatment or genetic manipulation have been demonstrated to be beneficial for transplant longevity (16). Hsps promote refolding of proteins denatured due to IRI, protecting cells from IRI-induced death. Hsp70 has been proposed to be the most potent anti-apoptotic mediator inside the cell (17). Heat pre-conditioning of organs prior to transplant upregulates the expression of Hsps and prevents tissue damage from IRI by different mechanisms (18). Hsps’ cytoprotective capacity was also demonstrated in organs that were genetically modified to overexpress these proteins. Hearts from mice overexpressing Hsp27 induction correlated with increased survival when transplanted in fully MHC-mismatched hosts (16). These hearts presented reduced caspase activation after subjection of ischemic/reperfusion conditions. In addition to heat, hypoxic pre-conditioning seems to reduce ischemic renal failure through a HIF-α/Hsp70 signaling pathway (19). This literature has been extensively reviewed in previous works (15, 20, 21).

## Hsps Can Protect from IRI by Modulating Inflammation

Recently, several studies have highlighted a previously overlooked importance of innate cells in shaping T cell-mediated responses to alloantigens (5). Indeed, IRI and the subsequent intra-organ activation of innate cells have been shown to markedly enhance alloimmunity, contributing to poorer long-term outcomes and graft function. For example, delayed graft function (DRF) is a complication that occurs very early after the transplant procedure and results from a previous intense ischemic injury. Kidney transplant patients with DRF have a higher risk to graft loss (22). Thus, strategies and treatments that prevent or decrease the activation of APCs by the released of ischemic-derived DAMPs could result in diminished alloimmunity and improve both early and late graft function (23).

During IRI, an important DAMP released by injured cells is the nucleotide adenosine triphosphate (ATP). Extracellular ATP (eATP) is recognized by purinergic receptors expressed by immune cells. Once eATP engages such receptors, it can trigger innate inflammatory responses and activation and proliferation of T cells. This can lead to further inflammation and cell damage, contributing to rejection [extensively reviewed in Ref. (24, 25)]. Additionally, high-mobility group box 1 (HMGB1) can also be released from dying cells. HMGB1 has been reported to be involved in IRI. HMGB-1 can activate APCs through TLR2 and TLR4 (26), as well as the receptor for advanced glycation end products (RAGE) (27), triggering anti-donor T cell responses (28).

Heat shock proteins have been suggested to act as DAMPs (29). Initial observations demonstrated that Hsps are elevated in transplanted organs, and Hsp-reactive T cells do infiltrate organs undergoing rejection (30). This raised the initial idea that such proteins play a crucial role as immunogenic antigens during alloimmune responses (15). Hsps are among many intracellular proteins that are released to the extracellular environment as a consequence of cell death during IRI. This is one reason why many consider Hsps to be DAMPS. Another reason is that extracellular isoforms of Hsps were reported by some studies to interact with TLRs and SRs and trigger inflammatory responses (31).

Aside from being passively released, Hsps can reach the extracellular milieu through different active pathways. Hsp70 can be exported by an active non-classical secretory pathway, which cannot be blocked by inhibitors of the ER–Golgi pathway (32). Also, Hsp70 can be released by a lysosome–endosome mechanism, similar to IL-1β secretion (33), and a pathway involving secretory-like granules (34). Finally, Hsp70 can be secreted by a mechanism involving the insertion into exosome membranes (35).

Thus, a scenario in which Hsps are found extracellularly during transplantation is likely, independently of passive or active release. More recent studies suggested that extracellular Hsps in transplants play additional immune roles – triggering anti-inflammatory responses and acting as immune modulators. In contrast to the DAMPs hypothesis, it was proposed that Hsps could belong to a group of molecules denominated resolution-associated molecular patterns (RAMPs) (36). RAMPs are released from necrotic and damaged cells, and when they reach the extracellular environment, will exert anti-inflammatory and regulatory effects over immune cells.

Resolution-associated molecular patterns are proposed to counterbalance acute inflammation and restore immune homeostasis by modulating innate cells. After tissue damage, they can modulate acute inflammation by inducing the production of IL-10 (36). IL-10 has powerful anti-inflammatory and immune suppressive properties. It can modulate DCs activation and differentiation, inhibits the release of inflammatory cytokines by T cells, APCs, and NK cells, and impairs cytotoxic ability of CD8 T cells (37). The alpha B-crystallin (αBC) protein is considered a RAMP. Mice deficient for this protein have an exacerbated form of experimental autoimmune encephalomyelitis (EAE). Administration of αBC to mice with EAE reduces severity disease scores (38). Interestingly, and maybe not coincidentally, αBC is a chaperone, and a member of the small Hsps group. The induction of IL-10 by other Hsps, such as Hsp70 (39), Hsp60 (40), and BiP (41), is well documented and has been reviewed elsewhere.

## Transplant of Organs Genetically Modified to Overexpress Hsps Generate Less Inflammation

A member of the Hsp70 family that has immunomodulatory effects and acts as a RAMP is the endoplasmic reticulum (ER) protein GRP78 or BiP (38). When islet cells overexpressing GRP78 were transplanted in fully MHC-mismatched hosts, they presented decreased cell death, prolonged survival, and were less immunogenic compared with controls (42). In a murine cardiac transplant model, transgenic Hsp27 overexpression increased allograft survival. Hsp27tg-derived hearts exhibited reduced IRI-induced apoptosis *ex vivo* and stimulated a reduced allogeneic inflammatory response compared with hearts transplanted from littermate controls (16). Markers for infiltrating T cells were reduced within transplanted hearts from Hsp27tg mice, and this was coupled with less production of IFN-γ day 5 post-transplant and increased IL-4 at day 2. In other inflammatory models, overexpression of Hsps has also been shown to be beneficial not only due to their pro-survival roles but also by dampening inflammation. Overexpression of Hsp70 in transgenic (Tg) mice has been shown to protect animals from neuroinflammation (43). Hsp70 overexpression can also induce neuroprotection from stroke and traumatic brain injury (44, 45). Consistent with Hsp70 possessing protective properties against tissue injury, Hsp70 Tg mice were found to have protection from inflammatory colitis and pulmonary fibrosis in respective models compared with their wild-type counterparts (46, 47).

In summary, increased levels of Hsps can be beneficial through the prevention of cell death, precluding the release of DAMPs. It is also possible that in HspTg mice, after tissue damage is induced by transplantation, the concentration of extracellular Hsps released from damaged cells is higher, resulting in induction of tolerogenic responses and the dampening of inflammation.

## Hsp Peptides as Antigens for Treg Cells

Regulatory T cells (Tregs) can suppress excessive effector immune responses that are harmful to the host (48). Tregs can actively suppress innate and adaptive inflammatory immune responses through the production of the anti-inflammatory cytokines IL-10 and/or TGF-β (49). Induction or administration of Tregs during transplantation is a promising approach for the management of allograft rejection (50). Although the peptide ligands for Tregs have not extensively been characterized, several studies have reported that they recognize self-peptides bound to MHC class II molecules (51). Tregs originate in the thymus (tTregs) but can also be induced at peripheral sites (pTregs) (52). For example, pTregs can be generated in a tolerogenic microenvironment by interacting with DCs producing anti-inflammatory cytokines and low levels of MHC II and co-stimulatory molecules (53).

Recently, it was shown that heat pre-conditioning of the organ had protective effects in acute kidney injury induced by IRI, and that protection was mediated by a direct immunomodulatory response of Hsp70-specific Tregs (54).

## Modulation of Allograft Rejection by Extracellular Hsps

In addition to benefits for transplant organs conferred by increased intracellular Hsp levels, a number of studies have now demonstrated various extracellular Hsps to also extend graft survival. For example, subcutaneous treatment of recipients prior to transplant with a single dose of full-length murine Hsp60 or with two of its peptides (p12 and p277) was found to prolong skin graft acceptance (55). Interestingly, no improvement in skin allograft survival was observed when recipients were treated with an Hsp60 peptide from *Mycobacterium tuberculosis* (55). The authors suggested the differential effect between these Hsp60 species was likely due to a shift from an IFN-γ- to IL-10-producing phenotype in self-Hsp60-specific T cells, a shift which was not induced by treatment with mycobacterial Hsp60 (56). Consistent with skin graft protection conferred by mouse Hsp60 peptide administration, intranasal pre-treatment with encapsulated human Hsp60-derived peptide (p277) increased skin graft survival in two minor mismatched mice models (57). In this study, treatment with human Hsp60 also induced production of the anti-inflammatory cytokine Il-10. Together the findings from these studies indicate that Hsp-mediated extension of graft survival may be closely related to IL-10 induction.

Heat shock protein 10 is an Hsp60 co-chaperone and was first described as early pregnancy factor (EPF) (58). This protein is found in pregnant women’s sera, and was described to be immunosuppressive, involved in fetus tolerance (58). The subcutaneous *in situ* delivery of recombinant Hsp10 improved skin allograft survival in rats (59). The authors suggested that Hsp10 would inhibit Th1 responses through donor DCs modulation (59).

Heat shock protein 70 (DnaK) from *M. tuberculosis* can improve graft survival in two different models of skin allografts. First, when the allogeneic B16F10 melanoma cells (H-2^b^/I-A^b^) are subcutaneously injected in BALB/c hosts (H-2^d^/I-A^d^), they are rejected due to MHC disparity. However, when those cells were injected in the presence of DnaK, they could form tumors in the hosts (60). *In situ* analysis demonstrated a tolerogenic environment with an increased infiltration of Tregs in DnaK-treated tumors, and depletion of Tregs abrogated DnaK-mediated tumor protection. Extracellular DnaK treatment of bone marrow-derived macrophages (BMMs) was also found to promote the immunosuppressive M2-like macrophage phenotype and favor tumor growth in a murine melanoma model (61). Together, these studies demonstrated DnaK to have immunosuppressive effects upon multiple cell types. To exclude that the extended graft acceptance observed upon DnaK pre-treatment was due to other tumor mechanisms of immune evasion, we tested whether DnaK pre-treatment *in situ* impacted upon alloreactive responses in a fully MHC-mismatched skin graft model. We observed that DnaK-treated allografts had a significant increase in survival when compared with controls and in addition, this effect was dependent on Tregs (60, 62).

In addition to Hsp60 and Hsp70 family members, Hsp90 proteins have also been shown to have protective properties. For example, subcutaneous treatment with mouse gp96 was shown to delay skin allograft rejection in minor and major mismatch models (63). In another study, intradermal treatment of heart transplanted rats with high doses of liver-purified gp96 from the donor strain prolonged graft survival. Treatment with gp96 appeared to improve cardiac graft function immediately post-transplantation. Interestingly, this treatment did not have an effect on graft survival if gp96 was derived from the host strain (64). The author’s proposed gp96 acted upon innate cells such as APCs, which led to a reduced T cell response and delayed rejection. As the delayed rejection effect was only observed when donor strain-derived gp96 was used, we would suggest that it was donor cells that were subject to the immune-suppressive properties of gp96. The author did not exclude the gp96 effects upon graft longevity could be due to its wound-healing properties (64).

## Conclusion and Perspectives

There is now substantial evidence to demonstrate the immunosuppressive potential of Hsps. These studies indicate that the anti-inflammatory properties of Hsps warrant further investigation into Hsp-based treatments for contexts in which repression of immune responses is desirable. As discussed here, Hsp treatments have been effective agents to inhibit alloimmunity and extend solid organ transplant survival in mice. One could hypothesize Hsp treatments that promote a tolerogenic environment to also have therapeutic applications for various autoimmune and inflammatory diseases.

It remains to be seen how universal the application of immune-regulatory properties of Hsps can be applied to transplants of different cells and tissues. It is also important to note that Hsps were reported to amplify inflammatory (31) and immune responses to tumor antigens (65). Thus, the impact of Hsps upon the resulting immune and inflammatory response currently appears to be very much context dependent (31).

**Figure 1:**
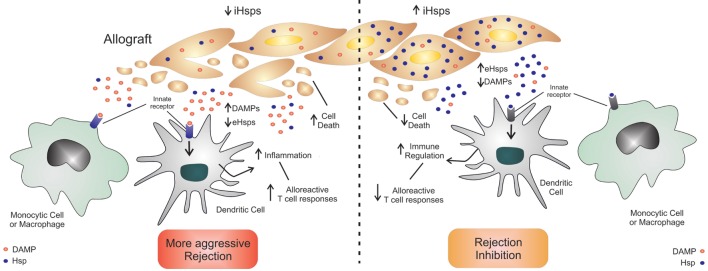
**Beneficial effects of Hsps during transplantation**. The upregulation of intracellular Hsps (iHsps) can protect cells from injury with a diminished release of DAMPs, decreased innate inflammation and less alloreactive T cell activation. The presence (in high concentrations) or administration of extracellular Hsps (eHsps) can also trigger immune-regulatory pathways on innate cells, favoring the generation of Tregs.

In solid organ transplantation contexts, however, most studies have indicated higher levels of intracellular and extracellular Hsps extend graft survival. This is likely due to a combination of the cytoprotective properties of Hsps enabling better survival following IRI and subsequent reduced DAMP release and inflammation as well as the immunomodulatory effect extracellular Hsps have upon multiple cell types including macrophages, T cells, and DCs. The resulting Hsp-induced immuno-biology described has included modulation of APCs to induce tolerogenic responses and regulatory T cells and decreased alloreactive T cell generation (Figure 1).

Thus, *ex vivo* manipulation of organs before the transplant in order to increase Hsps levels could constitute a promising approach in decreasing initial ischemic damage and inflammation, improving organ survival. Administration of Hsp-derived peptides or *ex vivo* expansion of Treg with Hsp-derived peptides could be an alternative strategy to improve solid organ outcome. Elucidation of the innate cell subsets and the receptors by which Hsps can specifically interact with will be extremely important to optimize Hsp-based therapy design.

## Author Contributions

TB, BL, RL, and CB wrote the paper. TB did the cartoon.

## Conflict of Interest Statement

The authors declare that the research was conducted in the absence of any commercial or financial relationships that could be construed as a potential conflict of interest.

## References

[1] Bianchi ME. DAMPs, PAMPs and alarmins: all we need to know about danger. J Leukoc Biol (2007) 81:1–5. 10.1189/jlb.0306164 17032697

[2] Matzinger P. The danger model: a renewed sense of self. Science (2002) 296:301–5. 10.1126/science.1071059 11951032

[3] Seong SY Matzinger P Hydrophobicity: an ancient damage-associated molecular pattern that initiates innate immune responses. Nat Rev Immunol (2004) 4:469–78. 10.1038/nri1372 15173835

[4] Jo SK Sung SA Cho WY Go KJ Kim HK. Macrophages contribute to the initiation of ischaemic acute renal failure in rats. Nephrol Dial Transplant (2006) 21:1231–9. 10.1093/ndt/gfk047 16410269

[5] Ochando J Kwan WH Ginhoux F Hutchinson JA Hashimoto D Collin M. The mononuclear phagocyte system in organ transplantation. Am J Transplant (2016) 16:1053–69. 10.1111/ajt.13627 26602545

[6] Otterbein LE Fan Z Koulmanda M Thronley T Strom TB. Innate immunity for better or worse govern the allograft response. Curr Opin Organ Transplant (2015) 20:8–12. 10.1097/MOT.0000000000000152 25563986 PMC4374347

[7] Farrar CA Kupiec-Weglinski JW Sacks SH. The innate immune system and transplantation. Cold Spring Harb Perspect Med (2013) 3:a015479. 10.1101/cshperspect.a015479 24086066 PMC3784815

[8] Li CY Lee JS Ko YG Kim JI Seo JS. Heat shock protein 70 inhibits apoptosis downstream of cytochrome c release and upstream of caspase-3 activation. J Biol Chem (2000) 275:25665–71. 10.1074/jbc.M906383199 10806214

[9] Ravagnan L Gurbuxani S Susin SA Maisse C Daugas E Zamzami N Heat-shock protein 70 antagonizes apoptosis-inducing factor. Nat Cell Biol (2001) 3:839–43. 10.1038/ncb0901-839 11533664

[10] Paul C Manero F Gonin S Kretz-Remy C Virot S Arrigo AP. Hsp27 as a negative regulator of cytochrome c release. Mol Cell Biol (2002) 22:816–34. 10.1128/MCB.22.3.816-834.2002 11784858 PMC133538

[11] Chauhan D Li G Hideshima T Podar K Mitsiades C Mitsiades N Hsp27 inhibits release of mitochondrial protein Smac in multiple myeloma cells and confers dexamethasone resistance. Blood (2003) 102:3379–86. 10.1182/blood-2003-05-1417 12855565

[12] Gurbuxani S Schmitt E Cande C Parcellier A Hammann A Daugas E Heat shock protein 70 binding inhibits the nuclear import of apoptosis-inducing factor. Oncogene (2003) 22:6669–78. 10.1038/sj.onc.1206794 14555980

[13] Richter K Haslbeck M Buchner J The heat shock response: life on the verge of death. Mol Cell (2010) 40(2):253–66. 10.1016/j.molcel.2010.10.006 20965420

[14] Verghese J Abrams J Wang Y Morano KA Biology of the heat shock response and protein chaperones: budding yeast (Saccharomyces cerevisiae) as a model system. Microbiol Mol Biol Rev (2012) 76(2):115–58. 10.1128/MMBR.05018-11 22688810 PMC3372250

[15] Pockley AG Muthana M. Heat shock proteins and allograft rejection. Contrib Nephrol (2005) 148:122–34. 10.1159/000086057 15912031

[16] Seemampillai B Germack R Felkin LE Mccormack A Rose ML. Heat shock protein-27 delays acute rejection after cardiac transplantation: an experimental model. Transplantation (2014) 98:29–38. 10.1097/TP.0000000000000170 24879379 PMC4164282

[17] Jaattela M. Heat shock proteins as cellular lifeguards. Ann Med (1999) 31:261–71. 10.3109/07853899908995889 10480757

[18] Jones Q Voegeli TS Li G Chen Y Currie RW. Heat shock proteins protect against ischemia and inflammation through multiple mechanisms. Inflamm Allergy Drug Targets (2011) 10:247–59. 10.2174/187152811796117726 21539516

[19] Yeh CH Hsu SP Yang CC Chien CT Wang NP. Hypoxic preconditioning reinforces HIF-alpha-dependent HSP70 signaling to reduce ischemic renal failure-induced renal tubular apoptosis and autophagy. Life Sci (2010) 86:115–23. 10.1016/j.lfs.2009.11.022 19962996

[20] O’Neill S Ross JA Wigmore SJ Harrison EM. The role of heat shock protein 90 in modulating ischemia-reperfusion injury in the kidney. Expert Opin Investig Drugs (2012) 21:1535–48. 10.1517/13543784.2012.713939 22876854

[21] O’Neill S Harrison EM Ross JA Wigmore SJ Hughes J. Heat-shock proteins and acute ischaemic kidney injury. Nephron Exp Nephrol (2014) 126:167–74. 10.1159/000363323 24923736

[22] Yarlagadda SG Coca SG Formica RN Poggio ED Parikh CR. Association between delayed graft function and allograft and patient survival: a systematic review and meta-analysis. Nephrol Dial Transplant (2009) 24:1039–47. 10.1093/ndt/gfn667 19103734

[23] Solhjou Z Athar H Xu Q Abdi R. Emerging therapies targeting intra-organ inflammation in transplantation. Am J Transplant (2015) 15:305–11. 10.1111/ajt.13073 25612486

[24] Vergani A Tezza S Fotino C Visner G Pileggi A Chandraker A The purinergic system in allotransplantation. Am J Transplant (2014) 14:507–14. 10.1111/ajt.12567 24433446

[25] Zeiser R Robson SC Vaikunthanathan T Dworak M Burnstock G. Unlocking the potential of purinergic signaling in transplantation. Am J Transplant (2016). 10.1111/ajt.13801 27005321 PMC5472988

[26] Yu M Wang H Ding A Golenbock DT Latz E Czura CJ HMGB1 signals through toll-like receptor (TLR) 4 and TLR2. Shock (2006) 26:174–9. 10.1097/01.shk.0000225404.51320.82 16878026

[27] Kokkola R Andersson A Mullins G Ostberg T Treutiger CJ Arnold B RAGE is the major receptor for the proinflammatory activity of HMGB1 in rodent macrophages. Scand J Immunol (2005) 61:1–9. 10.1111/j.0300-9475.2005.01534.x 15644117

[28] Land WG. Emerging role of innate immunity in organ transplantation part II: potential of damage-associated molecular patterns to generate immunostimulatory dendritic cells. Transplant Rev (Orlando) (2012) 26:73–87. 10.1016/j.trre.2011.02.003 22074784

[29] Chen GY Nunez G. Sterile inflammation: sensing and reacting to damage. Nat Rev Immunol (2010) 10:826–37. 10.1038/nri2873 21088683 PMC3114424

[30] Moliterno R Valdivia L Pan F Duquesnoy RJ. Heat shock protein reactivity of lymphocytes isolated from heterotopic rat cardiac allografts. Transplantation (1995) 59:598–604. 10.1097/00007890-199502270-00027 7878764

[31] Calderwood SK Gong J Murshid A. Extracellular HSPs: the complicated roles of extracellular HSPs in immunity. Front Immunol (2016) 7:159. 10.3389/fimmu.2016.00159 27199984 PMC4842758

[32] Hightower LE Guidon PT Jr. Selective release from cultured mammalian cells of heat-shock (stress) proteins that resemble glia-axon transfer proteins. J Cell Physiol (1989) 138:257–66. 10.1002/jcp.1041380206 2918030

[33] Mambula SS Calderwood SK. Heat shock protein 70 is secreted from tumor cells by a nonclassical pathway involving lysosomal endosomes. J Immunol (2006) 177:7849–57. 10.4049/jimmunol.177.11.7849 17114456

[34] Evdonin AL Martynova MG Bystrova OA Guzhova IV Margulis BA Medvedeva ND. The release of Hsp70 from A431 carcinoma cells is mediated by secretory-like granules. Eur J Cell Biol (2006) 85:443–55. 10.1016/j.ejcb.2006.02.008 16584808

[35] Vega VL Rodriguez-Silva M Frey T Gehrmann M Diaz JC Steinem C Hsp70 translocates into the plasma membrane after stress and is released into the extracellular environment in a membrane-associated form that activates macrophages. J Immunol (2008) 180:4299–307. 10.4049/jimmunol.180.6.4299 18322243

[36] Shields AM Panayi GS Corrigall VM. Resolution-associated molecular patterns (RAMP): RAMParts defending immunological homeostasis? Clin Exp Immunol (2011) 165:292–300. 10.1111/j.1365-2249.2011.04433.x 21671907 PMC3170978

[37] Moore KW De Waal Malefyt R Coffman RL O’Garra A. Interleukin-10 and the interleukin-10 receptor. Annu Rev Immunol (2001) 19:683–765. 10.1146/annurev.immunol.19.1.683 11244051

[38] Shields AM Thompson SJ Panayi GS Corrigall VM Pro-resolution immunological networks: binding immunoglobulin protein and other resolution-associated molecular patterns. Rheumatology (Oxford) (2012) 51:780–8. 10.1093/rheumatology/ker412 22190690

[39] Borges TJ Wieten L Van Herwijnen MJ Broere F Van Der Zee R Bonorino C The anti-inflammatory mechanisms of Hsp70. Front Immunol (2012) 3:95. 10.3389/fimmu.2012.00095 22566973 PMC3343630

[40] Quintana FJ Cohen IR The HSP60 immune system network. Trends Immunol (2010) 32:89–95. 10.1016/j.it.2010.11.001 21145789

[41] Shields AM Panayi GS Corrigall VM A new-age for biologic therapies: long-term drug-free therapy with BiP? Front Immunol (2012) 3:17 10.3389/fimmu.2012.00017 22566902 PMC3342250

[42] Wang M Wang P Liu YQ Peng JL Zhao XP Wu S The immunosuppressive and protective ability of glucose-regulated protein 78 for improvement of alloimmunity in beta cell transplantation. Clin Exp Immunol (2007) 150:546–52. 10.1111/j.1365-2249.2007.03525.x 17956578 PMC2219376

[43] Kim N Kim JY Yenari MA. Anti-inflammatory properties and pharmacological induction of Hsp70 after brain injury. Inflammopharmacology (2012) 20(3):177–85. 10.1007/s10787-011-0115-3 22246599

[44] Yenari MA Giffard RG Sapolsky RM Steinberg GK. The neuroprotective potential of heat shock protein 70 (HSP70). Mol Med Today (1999) 5:525–31. 10.1016/S1357-4310(99)01599-3 10562718

[45] Zheng Z Kim JY Ma H Lee JE Yenari MA. Anti-inflammatory effects of the 70 kDa heat shock protein in experimental stroke. J Cereb Blood Flow Metab (2008) 28:53–63. 10.1038/sj.jcbfm.9600502 17473852

[46] Tanaka K Namba T Arai Y Fujimoto M Adachi H Sobue G Genetic evidence for a protective role for heat shock factor 1 and heat shock protein 70 against colitis. J Biol Chem (2007) 282:23240–52. 10.1074/jbc.M704081200 17556362

[47] Tanaka K Tanaka Y Namba T Azuma A Mizushima T. Heat shock protein 70 protects against bleomycin-induced pulmonary fibrosis in mice. Biochem Pharmacol (2010) 80:920–31. 10.1016/j.bcp.2010.05.025 20513440

[48] Sakaguchi S Yamaguchi T Nomura T Ono M. Regulatory T cells and immune tolerance. Cell (2008) 133:775–87. 10.1016/j.cell.2008.05.009 18510923

[49] Vignali DA Collison LW Workman CJ. How regulatory T cells work. Nat Rev Immunol (2008) 8:523–32. 10.1038/nri2343 18566595 PMC2665249

[50] Tang Q Bluestone JA. Regulatory T-cell therapy in transplantation: moving to the clinic. Cold Spring Harb Perspect Med (2013) 3(11):a015552. 10.1101/cshperspect.a015552 24186492 PMC3808774

[51] Simons DM Picca CC Oh S Perng OA Aitken M Erikson J How specificity for self-peptides shapes the development and function of regulatory T cells. J Leukoc Biol (2010) 88:1099–107. 10.1189/jlb.0310183 20495071 PMC2996893

[52] Shevach EM Thornton AM. tTregs, pTregs, and iTregs: similarities and differences. Immunol Rev (2014) 259:88–102. 10.1111/imr.12160 24712461 PMC3982187

[53] Svajger U Rozman P. Tolerogenic dendritic cells: molecular and cellular mechanisms in transplantation. J Leukoc Biol (2014) 95:53–69. 10.1189/jlb.0613336 24108704

[54] Kim MG Jung Cho E Won Lee J Sook Ko Y Young Lee H Jo SK The heat-shock protein-70-induced renoprotective effect is partially mediated by CD4+ CD25+ Foxp3 + regulatory T cells in ischemia/reperfusion-induced acute kidney injury. Kidney Int (2014) 85:62–71. 10.1038/ki.2013.277 23884338

[55] Birk OS Gur SL Elias D Margalit R Mor F Carmi P The 60-kDa heat shock protein modulates allograft rejection. Proc Natl Acad Sci U S A (1999) 96:5159–63. 10.1073/pnas.96.9.5159 10220435 PMC21833

[56] Elias D Meilin A Ablamunits V Birk OS Carmi P Konen-Waisman S Hsp60 peptide therapy of NOD mouse diabetes induces a Th2 cytokine burst and downregulates autoimmunity to various beta-cell antigens. Diabetes (1997) 46:758–64. 10.2337/diab.46.5.758 9133541

[57] Luna E Postol E Caldas C Benvenuti LA Rodrigues JM Jr Lima K Treatment with encapsulated Hsp60 peptide (p277) prolongs skin graft survival in a murine model of minor antigen disparity. Scand J Immunol (2007) 66:62–70. 10.1111/j.1365-3083.2007.01951.x 17587347

[58] Rolfe BE Cavanagh AC Quinn KA Morton H. Identification of two suppressor factors induced by early pregnancy factor. Clin Exp Immunol (1988) 73:219–25. 3180511 PMC1541604

[59] Morton H Mckay DA Murphy RM Somodevilla-Torres MJ Swanson CE Cassady AI Production of a recombinant form of early pregnancy factor that can prolong allogeneic skin graft survival time in rats. Immunol Cell Biol (2000) 78:603–7. 10.1046/j.1440-1711.2000.00951.x 11114970

[60] Borges TJ Porto BN Teixeira CA Rodrigues M Machado FD Ornaghi AP Prolonged survival of allografts induced by mycobacterial Hsp70 is dependent on CD4+CD25+ regulatory T cells. PLoS One (2010) 5:e14264. 10.1371/journal.pone.0014264 21170379 PMC2999527

[61] Lopes RL Borges TJ Araujo JF Pinho NG Bergamin LS Battastini AM Extracellular mycobacterial DnaK polarizes macrophages to the M2-like phenotype. PLoS One (2014) 9:e113441. 10.1371/journal.pone.0113441 25419575 PMC4242626

[62] De Souza AP Bonorino C. Tumor immunosuppressive environment: effects on tumor-specific and nontumor antigen immune responses. Expert Rev Anticancer Ther (2009) 9:1317–32. 10.1586/era.09.88 19761435

[63] Kovalchin JT Mendonca C Wagh MS Wang R Chandawarkar RY. In vivo treatment of mice with heat shock protein, gp 96, improves survival of skin grafts with minor and major antigenic disparity. Transpl Immunol (2006) 15:179–85. 10.1016/j.trim.2005.07.003 16431284

[64] Slack LK Muthana M Hopkinson K Suvarna SK Espigares E Mirza S Administration of the stress protein gp96 prolongs rat cardiac allograft survival, modifies rejection-associated inflammatory events, and induces a state of peripheral T-cell hyporesponsiveness. Cell Stress Chaperones (2007) 12:71–82. 10.1379/CSC-237R.1 17441509 PMC1852895

[65] Weng D Song B Koido S Calderwood SK Gong J. Immunotherapy of radioresistant mammary tumors with early metastasis using molecular chaperone vaccines combined with ionizing radiation. J Immunol (2013) 191:755–63. 10.4049/jimmunol.1203286 23772032 PMC4085737

